# The nasal microbiota in health and disease: variation within and between subjects

**DOI:** 10.3389/fmicb.2015.00134

**Published:** 2015-03-02

**Authors:** Kristi Biswas, Michael Hoggard, Ravi Jain, Michael W. Taylor, Richard G. Douglas

**Affiliations:** ^1^Department of Surgery, The University of AucklandAuckland, New Zealand; ^2^School of Biological Sciences, The University of AucklandAuckland, New Zealand

**Keywords:** chronic rhinosinusitis, bacterial communities, 16S rRNA gene, bacterial abundance, bacterial diversity

## Abstract

Chronic rhinosinusitis (CRS) affects approximately 5% of the adult population in Western societies and severely reduces the patient’s quality of life. The role of bacteria in the pathogenesis of this condition has not yet been established with certainty. However, recent reports of bacterial and fungal biofilms in CRS highlight a potential role for these microorganisms. In this study, 16S rRNA gene-targeted amplicon pyrosequencing and qPCR were used to determine the composition and abundance, respectively, of the sinus microbiota within 9 patients with CRS and 6 healthy individuals. Within-patient variability was also investigated by sampling from anterior nares, inferior turbinate, and middle meatus on each side of the sinuses. Our results indicate that more of the variation in bacterial composition can be explained by inter-personal differences, rather than sampling location or even disease status. In addition, bacterial community diversity was significantly lower in CRS samples compared to those from healthy subjects, whereas bacterial load was not associated with disease status. Although members of the genera *Corynebacterium* and *Staphylococcus* were prevalent in the majority of samples (including healthy subjects), the large amount of variation observed between individuals, particularly within the CRS cohort, suggests that an imbalance or dysbiosis in community structure could be the driving force behind the disease. Ultimately, understanding the causes of variation within the sinus microbiota may lead to more personalized treatment options for CRS.

## INTRODUCTION

Chronic rhinosinusitis (CRS) is a persistent inflammatory condition of the nasal passages and the paranasal sinuses (Bhattacharyya, 2009; Soler et al., 2011). It affects approximately 5% of the Western population (Fokkens et al., 2012) and in the USA alone is responsible for an estimated $8.6 billion per annum in direct medical expenditures (Ray et al., 1999; Bhattacharyya, 2003). CRS results in significant morbidity in young and middle-aged adults, impacting substantially upon quality of life (Gliklich and Metson, 1995). Although antibiotics are used frequently in its treatment, the role of bacteria in the pathogenesis of this condition remains far from clear.

The role of changes in the nasal microbiota in the pathogenesis of CRS has come under increasing scrutiny, with a number of recent studies comparing the composition of bacteria in CRS patients vs. healthy subjects (Abreu et al., 2012; Feazel et al., 2012; Aurora et al., 2013; Boase et al., 2013). These studies, which have employed cultivation-independent (molecular) approaches to investigate the diversity of bacteria among samples, have yielded a range of results without a strongly emergent pattern. For example, the study of Abreu et al. (2012) utilized a 16S rRNA gene-targeting PhyloChip (diagnostic microarray; Brodie et al., 2006) to evaluate microbial community composition in CRS-affected and healthy individuals. The PhyloChip data implicated the bacterium *Corynebacterium tuberculostearicum* as a potential causative agent of CRS, particularly in conjunction with perturbation of the sinus microbiota. Importantly, this finding was supported by subsequent murine model experiments (Abreu et al., 2012). Other recent studies have linked CRS with changes in *Staphylococcus aureus* abundance or activity, decreased *Prevotella* spp., increased *Corynebacterium accolens*, lower microbial diversity, and increased microbial abundance, with no apparent consensus between individual studies (Feazel et al., 2012; Foreman et al., 2012; Boase et al., 2013; Ramakrishnan et al., 2013a; Choi et al., 2014; Cleland et al., 2014).

Considering the results of these recent studies, it is evident that the relationship between CRS and the sinus microbiota is complicated. The heterogeneous nature of the microbial association with CRS is highlighted by the marked differences in reported microbial communities between various studies, between phenotypic (polyps or without polyps; Fokkens et al., 2012) and immunological or histological subgroups of CRS (eosinophilic or neutrophilic; Kountakis et al., 2004; De Alarcón et al., 2006; Park et al., 2013; Bochenek et al., 2014) and the varying responses of patients to antibiotic and corticosteroid treatment (Joe et al., 2008; Liu et al., 2013). The causes of such variation are poorly understood, but could include differences among populations of patients (e.g., antibiotic history, ethnicity), methodology, genetics, environmental factors or simply natural variation among different parts of the nasal passages and sinuses.

Relatively few studies have investigated the impact of spatial variability in the microbiota of the human nasal cavity. One recent study found that epithelium type in healthy nasal passages has a significant impact on bacterial community diversity (Yan et al., 2013), with no distinct patterns in bacterial composition between sites. Another study demonstrated that the nasal microbiome is distinctly different to that of the oral and buccal cavity within an individual (Bassis et al., 2014). As both of these studies were conducted on healthy people, the impact of spatial variability in the nasal cavity of CRS patients is still not understood, let alone the extent of variation between different patients. It is a fundamental goal within microbial ecology to describe spatial variability in bacterial communities (Hanson et al., 2012), yet the clinical relevance of such research should also not be overlooked. Topical application of antibiotics or steroids, or even the addition of probiotic bacteria (Liu et al., 2013; Cleland et al., 2014), can be guided by knowledge of variation in bacterial composition and load within different parts of the nasal cavity. In short, an improved understanding of where and how the nasal microbiota varies should lead to improved, more personalized treatment options in the future.

In this study we used amplicon pyrosequencing and real-time PCR of the 16S rRNA gene to describe the composition and abundance of bacteria in subjects with and without CRS. Unlike many previous studies, we do not group CRS patients together but rather explore the differences in bacterial communities between each patient. Furthermore, we rigorously characterize within-subject variability in the nasal microbiota, with analysis of six samples per subject enabling novel insights into the spatial variation of these bacteria.

## MATERIALS AND METHODS

### PATIENT INFORMATION

Fifteen patients (nine CRS and six non-CRS controls) undergoing endoscopic sinus surgery were recruited for this study. The control group were undergoing pituitary tumor resection surgery. The CRS patients were chosen based on a Lund-Mackay score of >10/24 and the results of CT scans. Patient information is provided in **Table 1**. Written consent from the patients and ethical approval (NTX/08/12/126/AM01) from the New Zealand Health and Disability Ethics Committee, was obtained for this study.

**Table 1:** Patient information.

Patient No	Age	Ethnicity	Sex	Diagnosis	Asthma	Smoker	Prednisone*	Antibiotics*	L–M
1	46	NZE	M	CRS	Y	N	Y	N	11
2	43	NZE	M	CRS	N	N	N	N	15
3	51	NZE	F	CRS	Y	N	Y	N	20
4	48	NZE	M	CRS	Y	N	N	N	16
5	21	NZE	M	CRS, CF	N	N	N	N	21
6	27	Chile	F	CRS, polyposis	Y	N	N	N	14
7	69	other Euro	M	CRS	Y	N	N	N	15
8	39	Russian	M	CRS	Y/N	Y	Y	Y (Amoxicillin)	13
9	39	NZE	M	CRS, polyposis	N	N	N	N	13

**Patient No.**	**Age**	**Ethnicity**	**Sex**	**Diagnosis**	**Asthma**	**Smoker**	Prednisone*	Antibiotics*	**L–M**

1	53	NZE	M	Healthy	N	N	N	N	NA
2	80	NZE	M	Healthy	N	N	N	N	NA
3	65	other	M	Healthy	N	Y	N	N	NA
4	46	NZ Maori	F	Healthy	N	Y	N	N	NA
5	23	NZ Maori	F	Healthy	N	N	N	N	NA
6	31	Indian	M	Healthy	N	N	N	N	NA

NZE, New Zealand European; N, No; Y, Yes; CF, Cystic fibrosis; L –M, Lund–Mackay scale; Other, Asian ethnicity; Other Euro, Other European ethnicity. *Intake of Prednisone or antibiotics 4 weeks prior to surgery.

### SAMPLE COLLECTION

Surgery was performed on patients under general anesthetic. Sampling was performed immediately after induction prior to the application of any topical mucosal preparation or delivery of intravenous antibiotics. Sterile rayon-tipped swabs (Copan, #170KS01) were used under endoscopic guidance to sample the surface mucosa from each of three sites within the left and right sides of the nostril: anterior nares, inferior turbinate, and middle meatus. Duplicate swabs were taken from each of the six sites per patient, thus a total of 12 swabs obtained for each individual. Swabs were discarded and retaken if contaminated by mucosa outside the target region. Immediately after collection, the tip of each swab was removed aseptically and placed in a sterile 1.5 mL polypropylene tube on ice. Swabs were transported to the laboratory on ice within 2 h and stored at -20^∘^C until further analysis.

### DNA EXTRACTION

Two replicate swabs from each of the six sites were thawed on ice and placed together into a sterile Lysing Matrix E tube (MP Biomedicals, Australia). Genomic DNA was extracted from the samples using the AllPrep DNA/RNA Isolation Kit (Qiagen) following the manufacturer’s instructions and eluted in 30 μL of DNase-free water. Cells were ruptured using a Qiagen TissueLyser II at 25 m/s for 2 × 40 s. The quality and quantity of genomic DNA were measured on a Nanodrop 3300 fluorospectrometer and by using PicoGreen (Quant-iT dsDNA kit, Invitrogen) dye.

### 16S rRNA GENE AMPLICON PYROSEQUENCING

Bacterial 16S rRNA gene fragments from the extracted genomic DNA were amplified using primers 347f and 803r, which have been used previously to characterize the bacterial community of the human foregut (Nossa et al., 2010). The applicability of these primers to sinus microbial communities was validated *in silico* by using Probe Match in the RDP database and SILVA databases. Sample preparation for amplicon pyrosequencing was as described previously (Biswas et al., 2014), with some minor modifications. In brief, the aforementioned 16S rRNA gene-targeting primers, complete with pyrosequencing adaptors and unique multiplex identifiers (MIDs) on the forward primer, were used in equimolar concentrations (0.2 μM) together with dNTPs (0.2 mM), PCR buffer (1X), MgSO_4_ (2 mM), 0.5U Platinum Hi-fidelity Taq (Invitrogen) and PCR-certified water to a final volume of 25 μL. PCR amplification was performed in an Applied Biosystems Mastercycle gradient PCR machine with an initial denaturing step of 94^∘^C for 3 min, followed by 35 cycles of denaturation (94^∘^C for 30 s), annealing (55^∘^C for 30 s), and elongation (70^∘^C for 40 s), with a final elongation step at 70^∘^C for 3 min. Amplified products were purified using Agencourt AMPure beads (Beckman Coulter Inc.), quantified using Picogreen, and qualitatively checked on Agilent 1200 Bioanalyzer DNA 1000 chips (Agilent Technologies, Santa Clara, CA, USA). A nested-PCR approach, using primers 616V (Spring et al., 1998) and 1492R (Polz and Cavanaugh, 1998) for the initial amplification, was adopted for the samples of two CRS patient samples (2 and 3), as the original PCR did not yield any products.

Equimolar concentrations of 36 prepared amplicon samples were pooled into a single library in accordance with the instructions of Macrogen Inc. (Seoul, South Korea). Each amplicon library (three were required in total to analyze all 90 samples) was sequenced on 1/8 plate of the Roche GS FLX Titanium platform by Macrogen. Analysis of obtained pyrosequencing reads was carried out as described previously (Schmitt et al., 2012; Biswas et al., 2014). Briefly, a combination of mothur (Schloss et al., 2009) and custom-made PERL scripts was used to retain only high-quality useable reads that were aligned against the SILVA reference database (http://www.mothur.org/wiki/Silva_reference_alignment). Operational taxonomic units (OTUs) were assigned at 97% similarity based on an uncorrected pairwise distance matrix. A representative sequence from each OTU was subjected to taxonomic assignment using BLAST (Altschul et al., 1990) against a manually curated SILVA database (Version 108) using custom-made PERL scripts.

Multidimensional scaling (MDS) plots were constructed using Primer 6 software (version 6.1.6) of the 16S rRNA gene-based bacterial community composition at bacterial genus level. Bray–Curtis similarity was chosen as the measure between samples on the MDS plot.

### REAL-TIME PCR

The volume of sample obtained was variable across each site. Thus, to standardize the samples for quantification purposes across this study the relative proportions of human and bacterial DNA in the genomic extract was determined. For this purpose, human beta-actin genes were quantified using the previously described primers bactin-F (nucleotide position, 393–413) and bactin-R (622–642; Wang and Seed, 2003) targeting the *ACTB* gene. To quantify bacterial 16S rRNA gene abundance from extracted genomic DNA, we performed real-time PCR with primers 8F and 341R (Juck et al., 2000; Kim et al., 2009). Total numbers of 16S rRNA gene copies were calculated on the basis of the proportion of bacterial DNA for each extraction. Standards were prepared from clone libraries of bacterial 16S rRNA genes and from human genomic DNA (Promega) for *ACTB*. A 10-fold dilution series for each gene of interest was prepared (10–100,000 target copies per reaction for bacteria; 0.01–100 ng/μL for human DNA). Amplification efficiency of each primer set was calculated based on the respective standard curve using the formula: *E* = 10(^-1/slope^)-1 × 100%. Melting curve analysis was also performed to verify the specificity of the primer pairs, by using software ‘dissociation curve’ (Applied Biosystems). The reaction mix consisted of 10 μL of 1x QuantiTect SYBR Green master mix (QIAGEN) with HotStar Taq, 0.5 μl of each primer (10 μM), the respective genomic DNA template (10 ng for bacteria; 1 ng for human) or prepared standard and PCR grade water to a final volume of 20 μL. Thermal cycling conditions were as follows: 50^∘^C for 2 min, activation step at 95^∘^C for 15 min, followed by 40 cycles of denaturation (95 C for 15 s), annealing (60^∘^C for 1 min), and elongation (72^∘^C for 15 s). All samples including the non-template control and dilution series of standards were run in triplicate. Results were analyzed using the ABI Prism 7900HT sequence detection system (Version 2.4).

### STATISTICS

Diversity indices (including Shannon–Wiener index, Inverse-Simpson index, and rarefaction curves) were calculated on rarefied 16S rRNA gene sequence data for all samples at 97% similarity using mothur and then values were formally compared using Student’s *t*-test. In addition, equal numbers of sequences were subsampled to assess the significance of differences between sample types using UniFrac (phylogeny-based; Lozupone et al., 2011). Samples were assembled according to disease status, inter-personal differences and sampling site to test for the percentage of variation in samples accounted for by each group, as measured by *R*^2^, using permutational multivariate analysis of variance (PERMANOVA; Anderson, 2001). UniFrac (weighted and unweighted) distances calculated by mothur, along with the “Adonis” function of the vegan package in R software (Oksanen et al., 2013), were used for PERMANOVA. The obtained *R*^2^ values were then used to generate significance values (*p*-value) by comparison to 1000 random permutations of the data set.

## RESULTS

### MAJOR SOURCES OF VARIATION IN THE NASAL MICROBIOTA

**FIGURE 1:**
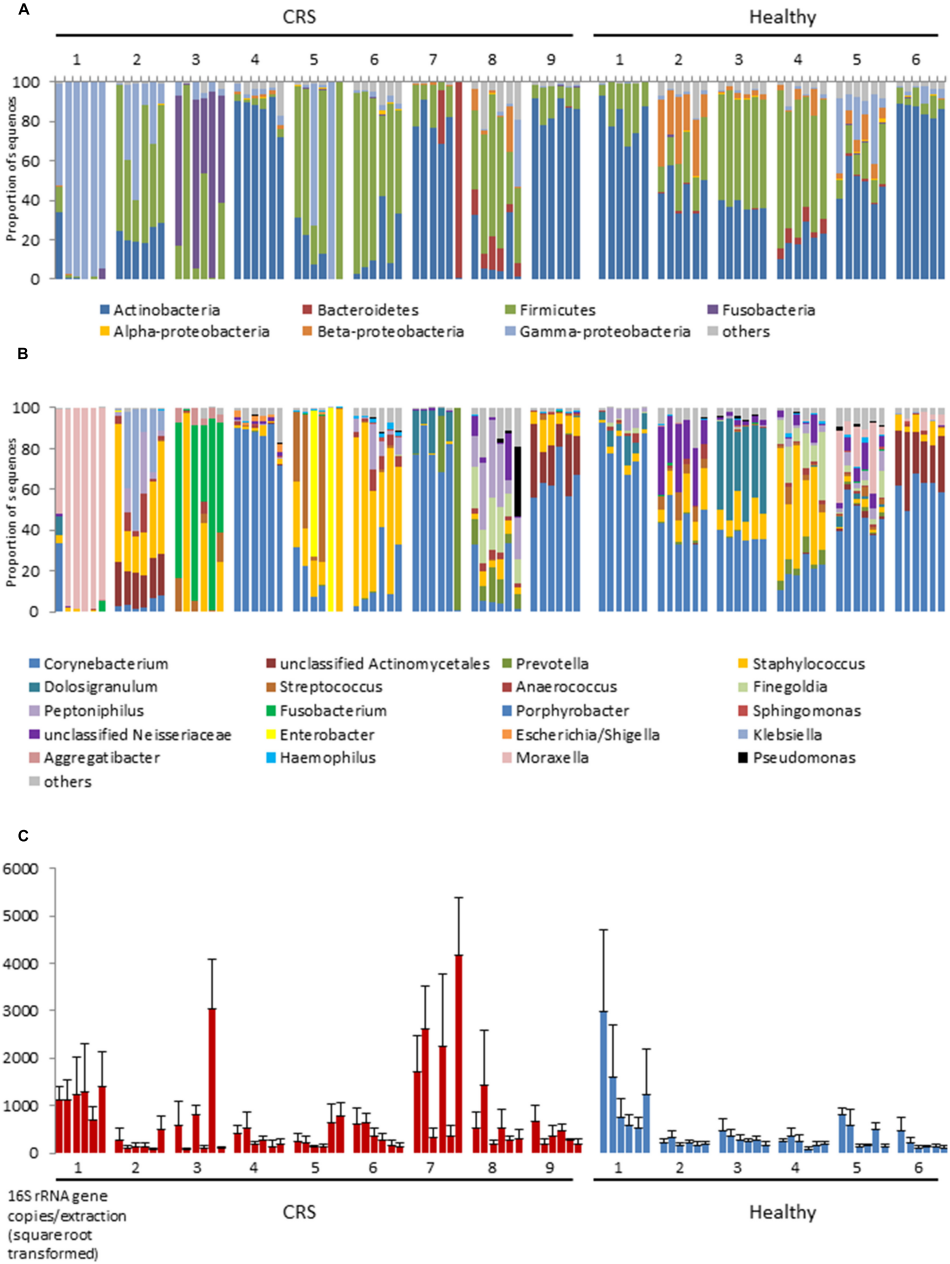
**16S rRNA gene-based bacterial community composition **(A,B)** and abundance **(C)** of nine CRS subjects and six healthy individuals.** Data representing each of six sites within the nasal cavities of each subject are shown. The order of the samples for each subject displayed is, from left to right: left anterior nares, right anterior nares, left inferior turbinate, right inferior turbinate, left middle meatus, right middle meatus. Bacterial community sequence data are displayed at phylum **(A)** and genus **(B)** levels, with data for each taxon expressed as a proportion of sequence reads for a given sample. 16S rRNA gene copy numbers are used as a proxy for bacterial abundance in qPCR, with bars indicating mean ± SD. (*n* = 3; **C**).

At bacterial phylum level the nasal microbiota has relatively low diversity (**Figure 1**). Most samples were dominated by members of the *Actinobacteria* (especially the genus *Corynebacterium*), *Firmicutes* (mostly *Staphylococcus* or *Dolosigranulum*), *Gammaproteobacteria* (especially *Moraxella*) and, in selected cases, *Fusobacteria*, or *Bacteroidetes*. Other phyla collectively comprised only a small proportion of the bacteria that were present. The sequence data summarized in **Figures 1A,B** reveals considerable variation among the microbiota of different individuals and, to a lesser extent, among different sites within a single individual. In order to formally partition this variation, Adonis was used to analyze the impact of disease status, inter-personal differences and sampling site on multi-species community structure of samples. The largest proportion of explained variation within our data was due to differences between individual patients (36.9%, *p* < 0.001), followed by disease status (3.8%, *p* < 0.001). Variation between sites of an individual, and between the left and right nostril, were also compared but these differences were not significant. It should be noted that the majority of the variation between samples (∼59%) was unexplained. Analyses of variance using Adonis based on weighted and unweighted UniFrac distance revealed similar outcomes, thus only unweighted UniFrac values are reported here. Below, each of these sources of variation is discussed.

### MICROBIOTA VARIATION AMONG DIFFERENT PATIENTS

**FIGURE 2:**
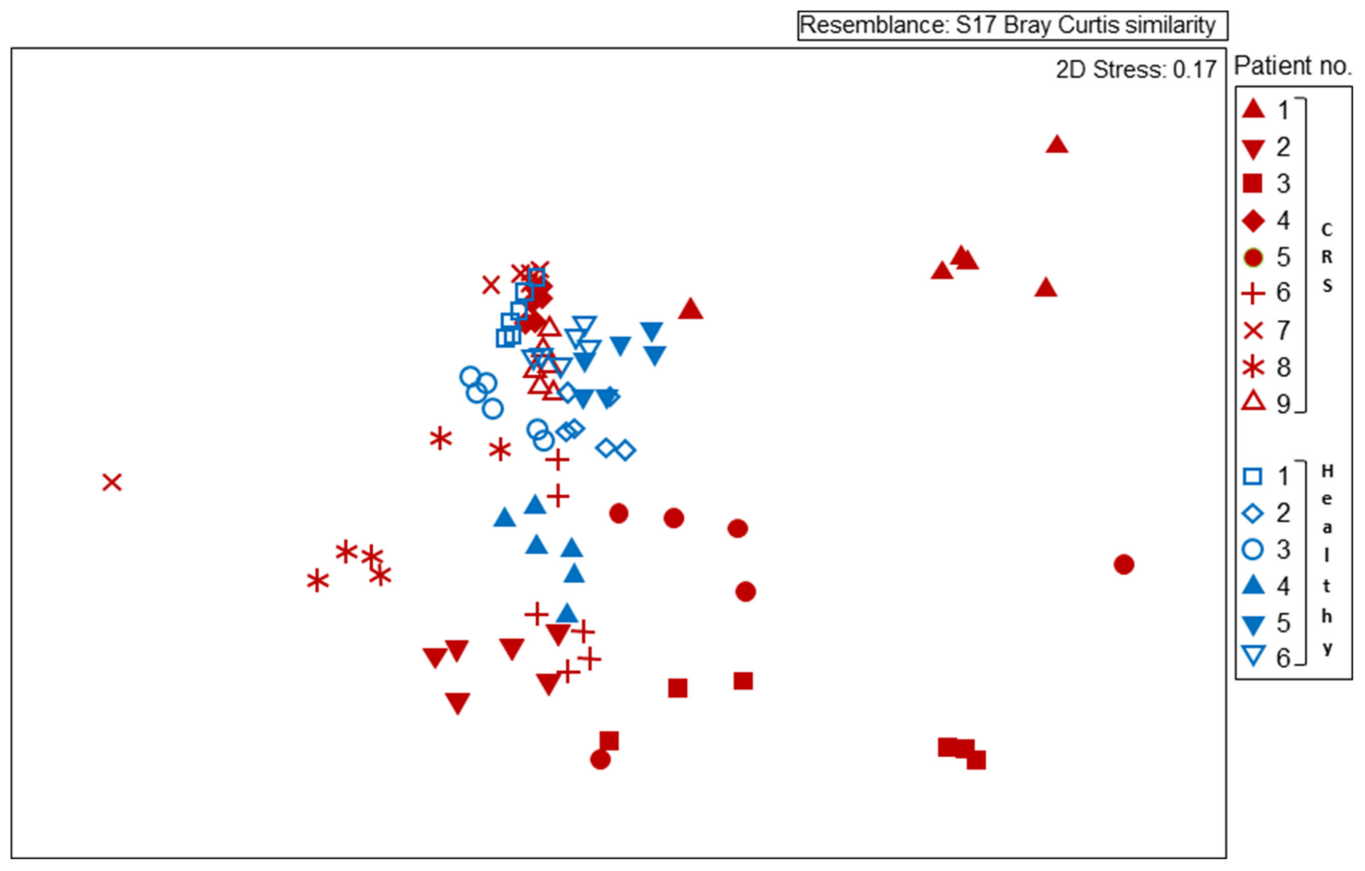
**Non-metric multidimensional scaling (MDS) plot displaying 16S rRNA gene-based bacterial community composition at bacterial genus level.** Subjects with CRS are signified by red symbols, with healthy subjects in blue symbols. Each subject is represented by six different samples, as outlined for **Figure 1**. Sequence data were compared among samples using Bray–Curtis similarity.

The largest source of variation in bacterial community composition could be attributed to differences among patients (36.9%). The microbiota varied considerably and non-predictably among the CRS patients, with a less variable microbiota being seen in the healthy subjects (**Figure 2**). Patients 3 and 4 from the healthy cohort were smokers and exhibited reduced *Actinobacteria* and increased *Firmicutes* sequence abundance compared with the other patients. However, such an obvious pattern was not observed with smoking among CRS cohort. Among the healthy cohort, members of the phyla *Actinobacteria*, *Firmicutes,* and, to a lesser extent, *Beta*- and *Gammaproteobacteria* dominated the communities, with these taxa represented largely by the genus *Corynebacterium* and other unclassified *Actinomycetales*, as well as *Peptoniphilus*, *Staphylococcus,* and *Moraxella* (**FigureS 1A,B**). The relative proportions of these main taxa varied substantially among the different healthy individuals. In contrast to their healthy counterparts, CRS patients displayed a more variable microbiota, with no consistent pattern emerging. CRS patients 4 and 9, for example, harbored a high proportion of *Actinobacteria* (especially *Corynebacterium*), with patient 7 also containing *Prevotella* (from the phylum *Bacteroidetes*) at some sinus sites. The gammaproteobacterium *Moraxella* dominated the sinus microbiota of CRS patient 1, but was not abundant in any of the other sampled CRS patients. *Staphylococcus* was abundant in multiple CRS (as well as healthy) patients, while *Fusobacterium* and *Streptococcus* were prevalent in patients 3 and 5, respectively. Unifrac (unweighted and weighted) and Adonis analyses identified significant differences between individuals (*p* < 0.001). Bacterial abundance, as estimated by the qPCR-based quantification of 16S rRNA gene copy numbers, varied among both CRS and healthy subjects (**Figure 1C**).

### INFLUENCE OF DISEASE STATUS ON THE NASAL MICROBIOTA

Disease status (whether or not a subject had CRS), accounted for only 3.8% of the variation observed within the bacterial community composition data. While there was no obvious, characteristic bacterial community “signature” associated with CRS, the microbiota of CRS sufferers did appear to be more variable than that of healthy subjects. Members of the genus *Corynebacterium*, which were abundant amongst all healthy subjects, only reached high abundance among CRS patients 4, 7, and 9, though they were present at lower numbers in some of the other CRS individuals. Ordination analyses (**Figure 2**) placed the bacterial communities of healthy subjects relatively close together, whereas the CRS-associated communities varied greatly, in some cases overlapping largely with the microbiota of healthy subjects and in other cases distinctly different. Clear subgroups of CRS microbiota were not evident. The abundance of bacterial 16S rRNA gene copies did not differ significantly between CRS patients and healthy subjects.

**FIGURE 3:**
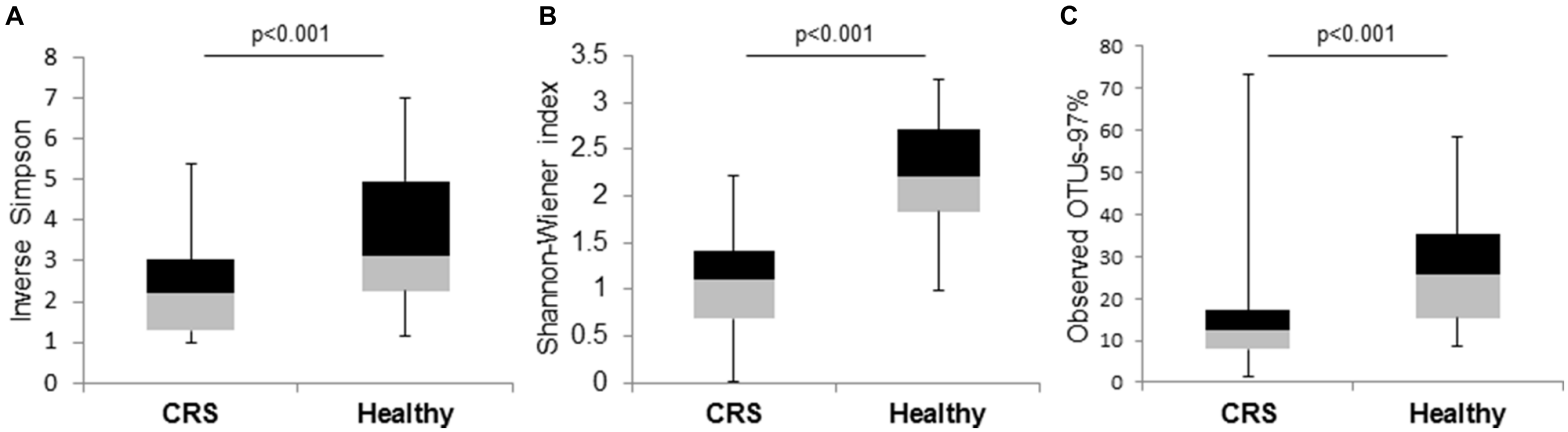
**Box and whisker plots to compare bacterial community diversity between CRS and healthy subjects. (A)** Inverse-Simpson, **(B)** Shannon–Wiener, and **(C)** observed OTUs-97% indices were calculated for the two groups of samples. Significant values (*p*-values) between the two groups are displayed on the plots.

The Shannon–Wiener, Inverse-Simpson, and observed OTUs (97%) diversity indices differed significantly (*p* < 0.001) between the microbial communities of the two cohorts of patients (**Figure 3**). These diversity analysis tools show that CRS patients exhibited lower bacterial diversity compared to their healthy counterparts.

### MICROBIOTA VARIATION AMONG DIFFERENT SITES WITHIN ONE PATIENT

Bacterial community composition and inferred abundance did not vary in a predictable manner across the six assayed sites within one patient (**Figure 1**). As indicated above, the microbiotas of CRS patients were particularly variable, even within some individuals. Only for patients 4, 6, and 9 were the communities relatively consistent for the six sample sites, in two cases dominated by *Corynebacterium* and in the third by *Staphylococcus*. Among the other diseased individuals, the microbiota of patient 1 was comprised almost exclusively of the gammaproteobacterium *Moraxella*, except for one nostril in which the anterior nares also contained a considerable amount of *Corynebacterium*. In contrast, bacterial communities at the six sites among the six healthy individuals remained fairly consistent at phylum and genus level. The bacterial load in patient 1 was significantly greater compared with those of the other five healthy individuals, but no obvious pattern was observed for any individual site within the nasal cavity. Bacterial diversity (measured with Shannon–Wiener, Inverse-Simpson, and observed OTUs-97%) did not differ significantly between sites for CRS and healthy individuals. The same applied to comparisons between nostrils.

## DISCUSSION

Although a number of molecular studies of the nasal microbiota have been published over the past decade, there has been no consensus on sample collection sites, analytical methods, or interpretation of the results obtained. Accordingly, comparison of results from different studies is difficult. Bacterial communities are known to have distinct biogeographic patterns within their environment (Hanson et al., 2012). However, there has been little research to investigate such variation within the human nasal cavities. In this study we investigated the spatial variability of the nasal microbiota both within an individual subject and between patients with CRS and normal subjects.

The high amount of unexplained variation observed in this study is typical of many data sets of ecological study (Borcard et al., 1992), where it is not feasible to measure all the environmental variables such as biological interactions and external environmental factors. The parameters that were specifically examined in this study accounted for only about 41% of the variability observed. In future studies, other factors that measure host interaction (such as cytokines) should also be considered along with the microbial component.

Small cohort sizes are an inherent limitation of studying CRS, as noted previously in the literature (Abreu et al., 2012; Feazel et al., 2012; Choi et al., 2014). The requirement for patients to remain off antibiotics for at least 4 weeks prior to surgery, coupled with the difficult nature of obtaining the samples (via endoscopic sinus surgery), inevitably results in small sample sizes. In addition, acquiring samples from healthy subjects is particularly difficult, as only patients undergoing FESS are recruited for the study. The cohort used in this study is small, but it is comparable to that in many other published studies and was still sufficient to give us significant results for comparisons between patients and disease status. Intra-patient variability was examined per patient, thus we speculate that a larger cohort would not change the outcome of this result.

### MICROBIOTA VARIATION AMONG DIFFERENT PATIENTS

This study indicates that disease status (CRS vs. healthy) accounted for less of the observed variation than inter-patient differences. These results are consistent with other human-associated microbiota studies on the gut (Turnbaugh et al., 2008), oral cavity (Nasidze et al., 2009), and skin (Grice et al., 2009), which reported high levels of variability among individuals. Another such study, investigating the overall human microbiota across different body parts of healthy subjects, also indicated that a personalized microbiota is relatively stable within a given individual over time (Costello et al., 2009) and that variation in microbial composition was largely due to inter-personal differences. Other studies on healthy- and CRS-associated nasal microbiota have also found large variation among individuals (Feazel et al., 2012; Ramakrishnan et al., 2013b). The results of this study build on upon the emerging understanding of CRS as a heterogeneous group of diseases that share clinical symptoms (Benninger et al., 2003; Van Crombruggen et al., 2011). These findings have implications for clinical treatment and prevention of CRS. It may be that treatment optimized to the specific microbiota of the patient is required.

It has been previously shown that smoking causes the relative proportion of *Firmicutes* to increase, and *Actinobacteria* to decrease, in healthy (non-CRS) individuals (Ramakrishnan et al., 2013b). The slight variations observed in the healthy cohort within our study might be explained by smoking, as this phenomenon was observed in patients 3 and 4 (both smokers) compared with the non-smokers. However, amongst the CRS cohort only one subject was a smoker. Thus, the considerable variability in bacterial composition between individuals of the CRS cohort still remains largely unexplained.

### EFFECT OF DISEASE STATUS ON THE NASAL MICROBIOTA

Bacterial abundance was measured in this study using 16S rRNA gene targeted qPCR. Again, the results were highly variable and not related to disease status. These results are consistent with the findings of others who used similar methodology (Abreu et al., 2012). However, other studies using different techniques to that used in this study have reported a significantly greater bacterial load (in particular *S. aureus*) in CRS patients compared with normal controls (Foreman et al., 2009; Boase et al., 2013).

These data indicate a greater degree of variation in microbial community structure among CRS patients compared to healthy individuals. However, the bacterial diversity of CRS patients was reduced. This finding has been observed in several other inflammatory diseases (e.g., cystic fibrosis, inflammatory bowel disease; Ott et al., 2004; Guss et al., 2010). The more stable community structure of healthy individuals suggest that dysbiosis (or imbalance) in the microbial community is linked to the occurrence of CRS. Microbial dysbiosis could also be due to the multiple courses of antibiotics prescribed to CRS patients in the years prior to surgery, which could in turn contribute to the lower bacterial diversity (Liu et al., 2013).

Another emerging view of CRS is that it is a fundamentally host immune system-mediated disease and requires sub-classification based on immunological and histological factors (Akdis et al., 2013). Further studies are required to clarify the role of the host immune-mediated responses in CRS and their interaction with microbial communities.

### SAMPLING WITHIN THE NASAL CAVITY

Microbial community structure did not differ significantly between sites or between the left and right nostrils of an individual. Of the four parameters chosen to investigate variation in samples, these two had the least impact. The slight variations observed in bacterial load or diversity, as seen in CRS patients 3, 5, and 7, could be due to the heterogeneous nature of the disease. The anterior naris is considered to be a relatively dry environment, with a different epithelium type compared to the other two sites analyzed in this study. The middle meatus and inferior turbinates are deeper in the nasal cavity and covered in a mucus blanket that is secreted by goblet cells. This provides an ideal environment for biofilm development, as observed previously in CRS patients (Cryer et al., 2004; Foreman et al., 2012). Based on the findings of Yan et al. (2013) and Bassis et al. (2014) and the different microenvironments of the study sites, we might have expected the nasal microbiota of the chosen sites to differ. However, our study detected no significant differences in bacterial diversity, composition or abundance between the different sites of either CRS or healthy individuals. Furthermore, different sides of the nasal cavity contained essentially the same microbiota, another important finding from this study. This study is the first of its kind for CRS and should be taken into consideration when designing future research in this area.

## CONCLUSION

This study provides some insight into the differences among microbial communities in the human nasal ecosystem. Somewhat unexpectedly, we observed that between-patient differences explained more of the variation in the nasal microbiota than did disease status or different sampling sites within the nasal cavities. An improved understanding of the causes of variation in bacterial composition, diversity, and abundance in the nasal cavity of CRS patients may help tailor improved clinical treatments in the future.

## Conflict of Interest Statement

The authors declare that the research was conducted in the absence of any commercial or financial relationships that could be construed as a potential conflict of interest.
